# Supplementary material for: Integrative genomic analysis reveals mechanisms of immune evasion in P. falciparum malaria
Source: Nat Commun. 2020 Oct 9;11:5093. doi: 10.1038/s41467-020-18915-6 (PMC7547729; doi:10.1038/s41467-020-18915-6)
Supplement: Supplementary file 1 — Supplementary Information [file 41467_2020_18915_MOESM1_ESM.pdf]

Supplementary Information

Integrative genomic analysis reveals mechanisms of immune evasion in *P. falciparum* malaria

Mame Massar Dieng[1†], Aïssatou Diawara[1†], Vinu Manikadan[1], Hala Tamim El Jarkass[1,6], Samuel Sindie Sermé[2], Salif Sombié[2], Aïssata Barry[2], Sam Aboubacar Coulibaly[2], Amidou Diarra[2], Nizar Drou[3], Marc Arnoux[4], Ayman Yousif[3], Alfred B. Tiono[2], Sodiomon B. Sirima[2,5], Issiaka Soulama[2] and Youssef Idaghdour[1*]

[1] Program in Biology, Division of Science and Mathematics, New York University Abu Dhabi, Abu Dhabi, United Arab Emirates. [2]Centre National de Recherche et de Formation sur le Paludisme, Ouagadougou, Burkina Faso. [3]Bioinformatics Core, New York University Abu Dhabi, Abu Dhabi, United Arab Emirates. [4]Core Technology Platforms, New York University Abu Dhabi, Abu Dhabi, United Arab Emirates. [5]Groupe de Recherche Action en Santé, Ouagadougou, Burkina Faso. [6]Present address: Department of Molecular Genetics, University of Toronto, Toronto, Ontario, Canada.

These authors contributed equally: Mame Massar Dieng, Aïssatou Diawara
[*]Correspondence to: youssef.idaghdour@nyu.edu

Content:

Supplementary Figures 1-5
List of Supplementary Tables 1-8

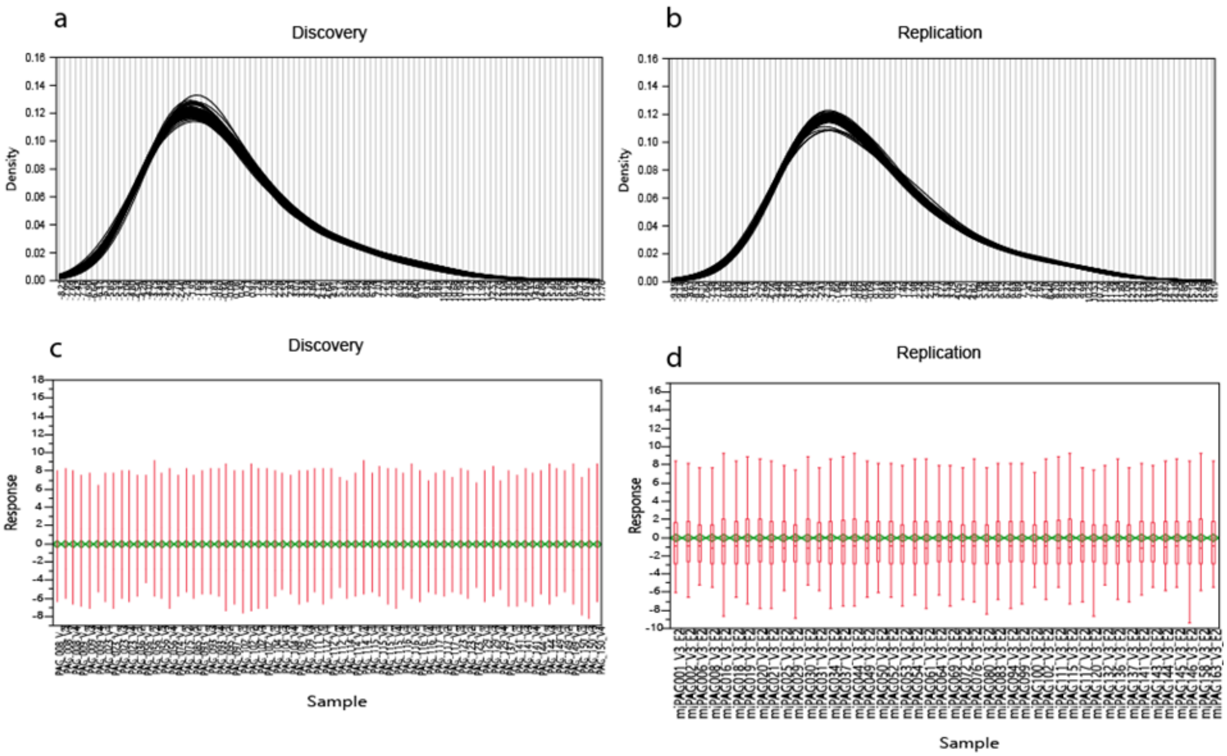

Supplementary Fig. 1 Distribution analysis of miRNA expression after Mean normalization. (**a**, **c**) and (**b**, **d**) correspond to the density and response of all miRNAs for each sample in the discovery (n = 68 samples: BI; n = 19, AP; n = 16, SP; n = 17, and AT; n = 16), and replication (n = 53) sets, respectively. The box plots in **c** and **d** show the median, the 25th and 75th percentiles as box edges, and the 5th and 95th percentiles as bounds of whiskers.

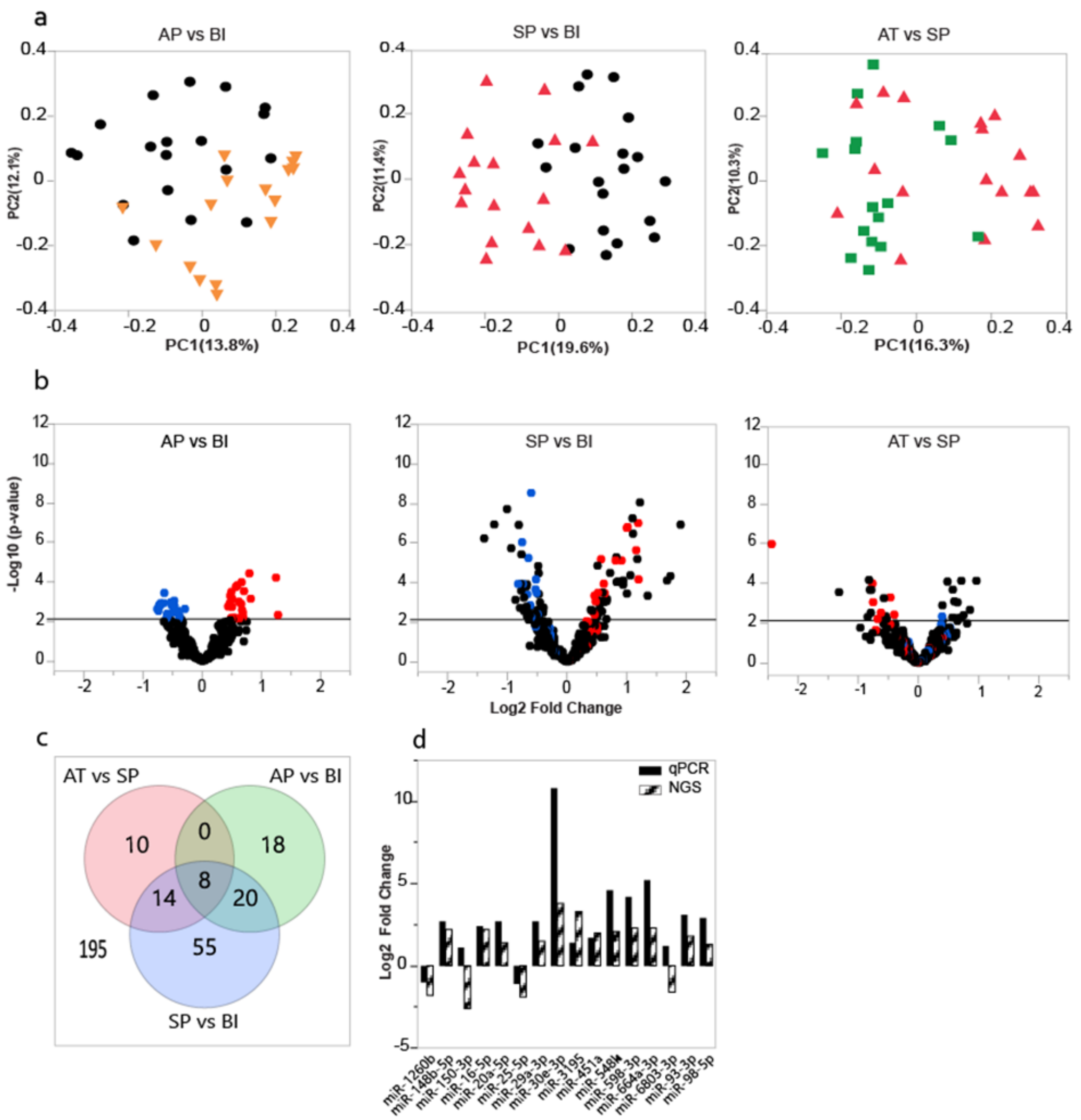

Supplementary Fig. 2 Principal Component Analysis (PCA) and differential expression of miRNA in the discovery set (n = 68). Panel **a** shows from left to right the PCA plots of miRNA expression profiles contrasting various infection stages: (Asymptomatic Parasitemia AP, yellow inverted triangles) vs (Before Infection BI, black dots), (Symptomatic Infection SP, red triangles) vs (BI), and (After Treatment AT, green squares) vs (SP). **b** Volcano plots of differential miRNA expression for the contrasts (from left to right): AP (n = 16) vs BI (n = 19), SP (n = 17) vs BI (n = 19), and AT (n = 16) vs SP (n = 17). Upregulated and downregulated miRNAs in the AP vs BI contract are labeled in red and blue and their pattern of differential expression is shown in the SP vs BI and AT vs SP contrasts. The magnitude (log2 fold change; x-axis) and significance (-log10 *P*) of differential expression of individual miRNA were obtained using repeated measures analysis of covariance (see Methods). The horizontal line indicates the -log10 *P* value corresponding to 5% Benjamini-Hochberg (B-H) FDR. **c** Venn diagram comparing the differentially expressed (B-H FDR $P < 0.05$) miRNA for the same three contrasts shown in **a** and **b**. **d** Bar plots representing the log2 Fold change between BI (n=20) and SP (19) of 16 miRNAs in the NGS analysis tested for qPCR validation.

Model Settings

Predictor Reduction Settings: Stat Test = Pearson, Multiple Testing Method = FDR, -log10(*P*) Cutoff = 1.3
Analysis Settings: Model Selection Method = Stepwise, Stop Criterion = AICC
Final Selected Variables: has-miR-199-3p, has-miR-3173-5p, has-miR-342-3p and has-miR-532-5p

---

Training Set Criteria: Root Mean Square Error = 1.4151, Harrell C-Statistic = 0.8185, Correlation = 0.8171

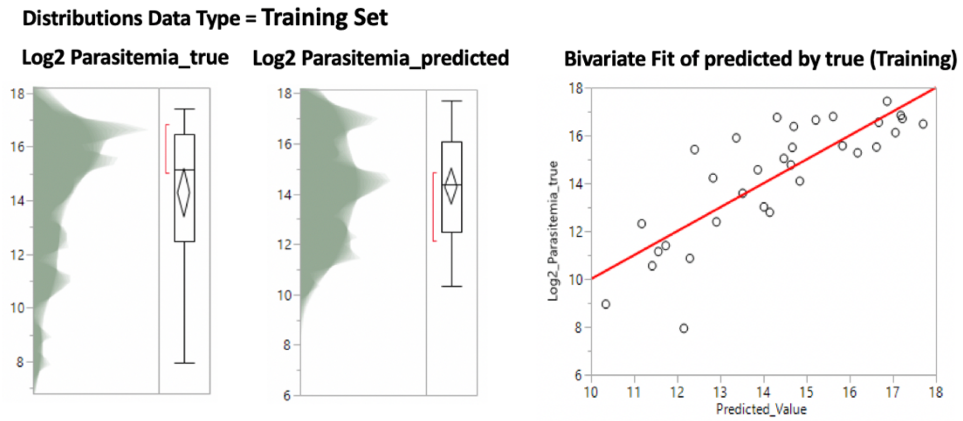

Test Set Criteria: Root Mean Square Error = 2.1983, Harrell C-Statistic = 0.8238, Correlation = 0.7339

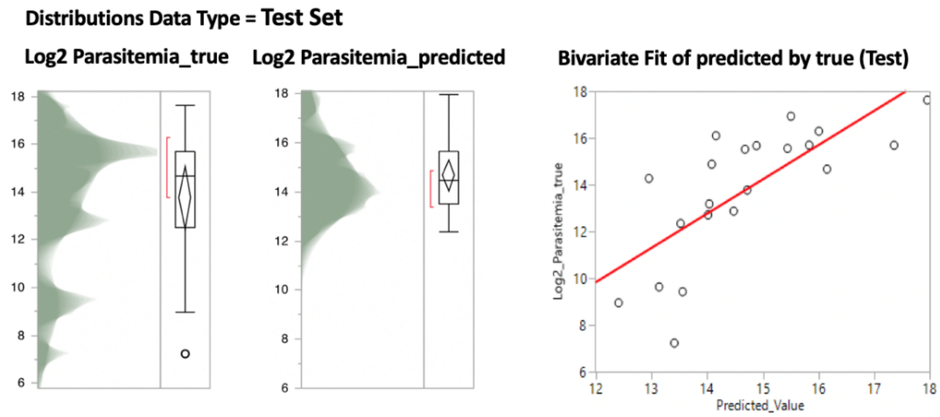

Supplementary Fig. 3 Prediction of log2 parasitemia using miRNAs expression levels. Predictive modeling was performed using SAS (SAS Institute). Predictor reduction was based on a B-H FDR threshold of 5% of Pearson correlation using a stepwise general linear model accounting for age, sex and white blood count and including all 72 miRNAs associated with parasitemia. The set of four miRNAs listed above were identified as the best predictive variables. Performance of the model was assessed using two-fold cross-validation done by randomly shuffling the dataset into training and test sets (60 and 40% of the full dataset, respectively) and using the following three metrics: Root Mean Squared Error, Harrell's C-Statistic and Pearson correlation. The distribution of the true and predicted log2 parasitemia values and their bivariate fit are shown for both the training (upper panel) and test (lower panel) sets. The data presented within each condition (discovery set: BI; n = 19, AP; n = 16, SP; n = 17, and AT; n = 16, and replication set; n = 53) are from biologically independent samples. The box plots in Figures **c** and **d** show the median, the 25th and 75th percentiles as box edges, and the 5th and 95th percentiles as bounds of whiskers.

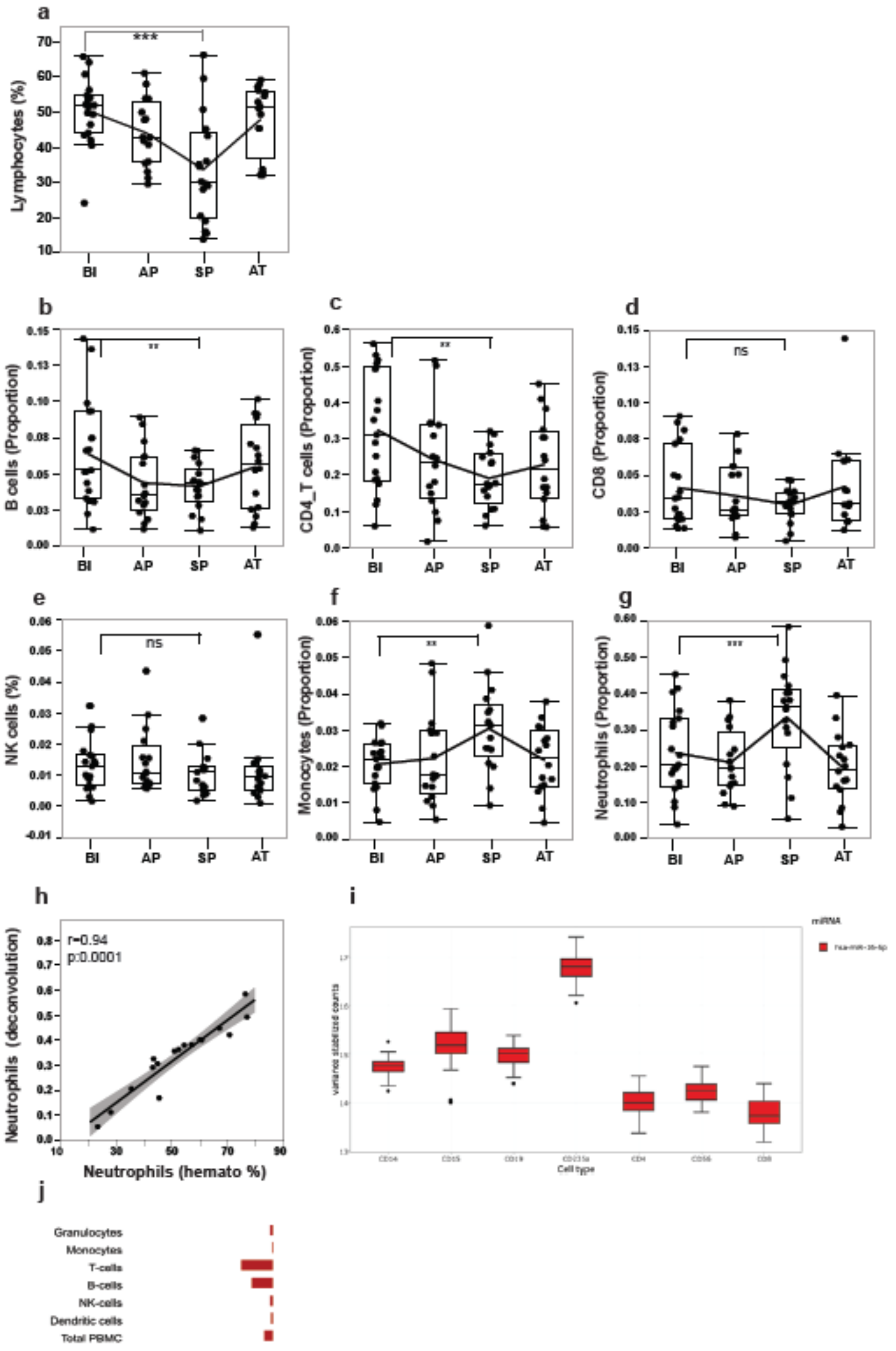
a
Lymphocytes (%)
***
BI
AP
SP
AT
b
B cells (Proportion)
**
c
CD4_T cells (Proportion)
**
d
CD8 (Proportion)
ns
e
NK cells (%)
ns
f
Monocytes (Proportion)
**
g
Neutrophils (Proportion)
***
h
r=0.94
p:0.0001
Neutrophils (deconvolution)
Neutrophils (hemato %)
i
miRNA
Cell type
j
Granulocytes
Monocytes
T-cells
B-cells
NK-cells
Dendritic cells
Total PBMC

Supplementary Fig. 4 Major immune cell types changes between the four stages of infection profiled. **a** The percentage of lymphocytes in the discovery set at the four stages of infection (n = 68: BI; n = 19, AP; n = 16, SP; n = 17, and AT; n = 16). Statistical significance of change in lymphocyte percentages between the BI and SP groups was assessed using two-tailed Student's t-test (*P* = 4e-5). **b-g** Proportion of sub-cell types in the discovery set (n = 68: BI; n = 19, AP; n = 16, SP; n = 17, and AT; n = 16) estimated using the tool Epic (Reference 31). Difference in the proportion of sub-cell type between the BI and SP groups was assessed using two-tailed Student's t-tests (B-cells ** *P* = 0.0192; CD4_T ** *P* = 0.0035; Monocytes ** *P* = 0.0064, Neutrophils ****P* = 0.0007, ns = non-significant). **h** Performance of estimation of the EPIC tool assessed by comparing the percentage of neutrophils measured using a blood cell analyzer instrument and the values estimated using the EPIC tool (n = 17, Pearson correlation coefficient r and *P* value are shown). The data presented within each condition (discovery set: BI, AP, SP and AT; replication set) are from biologically independent samples. **i** miR-16-5p RNASeq expression data retrieved from the BloodmiRs reference dataset of detailed miRNA expression profiles from seven types of human peripheral blood cells: CD56 (NK cells; n = 5), CD19 (B lymphocytes; n = 6), CD8 (cytotoxic T lymphocytes; n = 5), CD4 (T helper cells; n = 5), CD14 (monocytes; n = 7), CD15 (neutrophils; n = 6), and CD235 (erythrocytes; n = 6). **j** Consensus normalized expression levels of BCL2 gene for 6 blood cell types (granulocytes; n= 4, monocytes; n = 6, T-cells; n = 6, B-cells; n = 6, NK cells; n = 6, dendritic cells; n = 5, and total PBMCs; n = 6), created by combining the data from the three transcriptomics datasets (HPA, GTEx and FANTOM5, data retrieved from https://www.proteinatlas.org/ENSG00000171791-BCL2/tissue). The box plots in Figures **a**, **g** and **i** show the median, the 25th and 75th percentiles as box edges, and the 5th and 95th percentiles as bounds of whiskers.

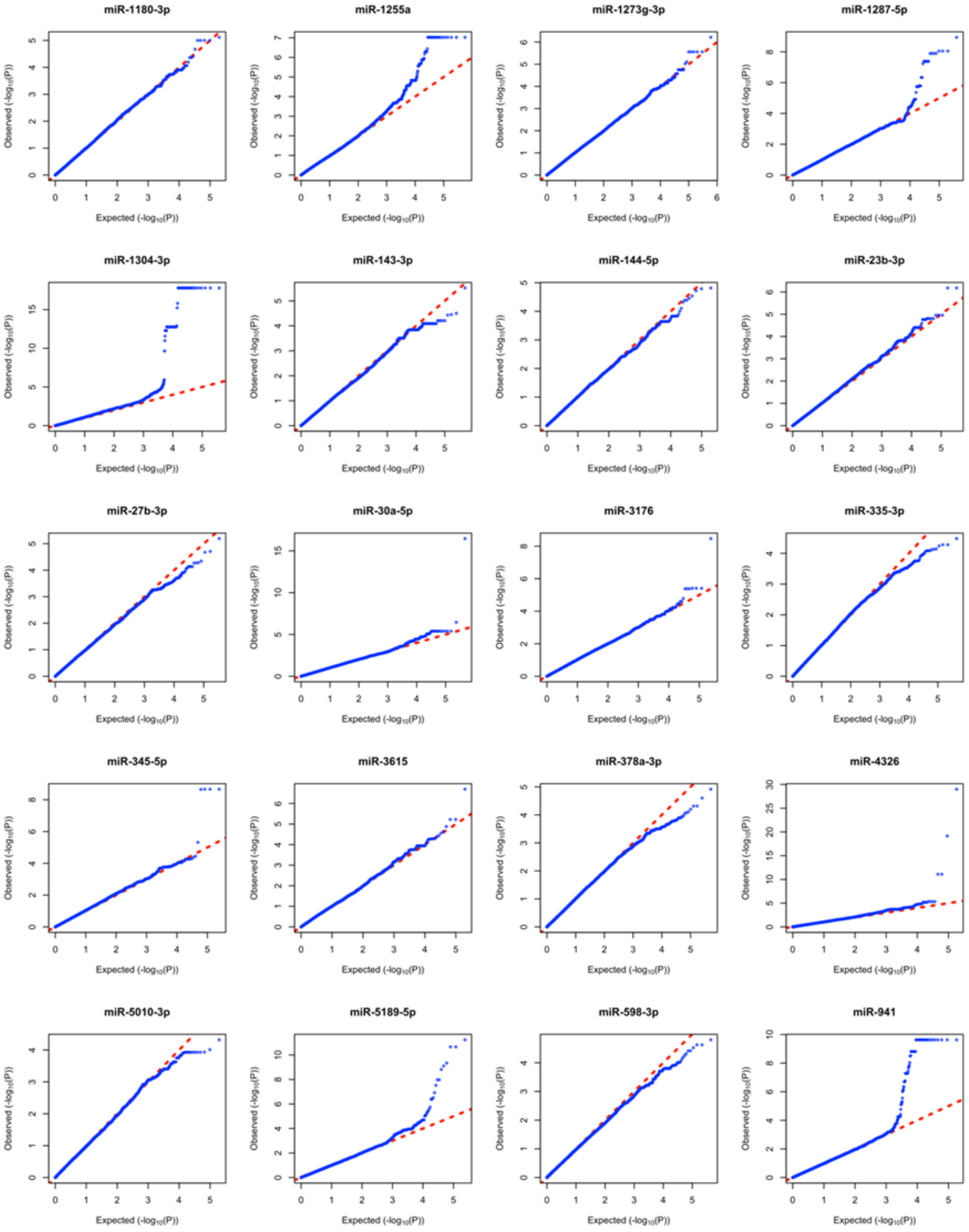

Supplementary Fig. 5 QQ plots for 20 significant eQTL-miRNA associations listed in Table 1. The -log10 of the observed nominal *P* value (multiple regression model in PLINK v1.9) of each miRNA-SNP association are plotted against expected values.

File Name: Supplementary Data 1

File Name: Supplementary Data 2

File Name: Supplementary Data 3

File Name: Supplementary Data 4

File Name: Supplementary Data 5

File Name: Supplementary Data 6

File Name: Supplementary Data 7

File Name: Supplementary Data 8
