# Supplementary material for: Integrative genomic analysis reveals mechanisms of immune evasion in P. falciparum malaria
Source: Nat Commun. 2020 Oct 9;11:5093. doi: 10.1038/s41467-020-18915-6 (PMC7547729; doi:10.1038/s41467-020-18915-6)
Supplement: Supplementary file 3 — Reporting Summary [file 41467_2020_18915_MOESM3_ESM.pdf]

nature research

Corresponding author(s): Youssef Idaghdour

Last updated by author(s): Sep 15, 2020

Reporting Summary

Nature Research wishes to improve the reproducibility of the work that we publish. This form provides structure for consistency and transparency in reporting. For further information on Nature Research policies, see our Editorial Policies and the Editorial Policy Checklist.

Statistics

For all statistical analyses, confirm that the following items are present in the figure legend, table legend, main text, or Methods section.

n/a	Confirmed	
☐	☒	The exact sample size (*n*) for each experimental group/condition, given as a discrete number and unit of measurement
☐	☒	A statement on whether measurements were taken from distinct samples or whether the same sample was measured repeatedly
☐	☒	The statistical test(s) used AND whether they are one- or two-sided<br>*Only common tests should be described solely by name; describe more complex techniques in the Methods section.*
☐	☒	A description of all covariates tested
☐	☒	A description of any assumptions or corrections, such as tests of normality and adjustment for multiple comparisons
☐	☒	A full description of the statistical parameters including central tendency (e.g. means) or other basic estimates (e.g. regression coefficient) AND variation (e.g. standard deviation) or associated estimates of uncertainty (e.g. confidence intervals)
☐	☒	For null hypothesis testing, the test statistic (e.g. *F*, *t*, *r*) with confidence intervals, effect sizes, degrees of freedom and *P* value noted<br>*Give P values as exact values whenever suitable.*
☒	☐	For Bayesian analysis, information on the choice of priors and Markov chain Monte Carlo settings
☒	☐	For hierarchical and complex designs, identification of the appropriate level for tests and full reporting of outcomes
☐	☒	Estimates of effect sizes (e.g. Cohen's *d*, Pearson's *r*), indicating how they were calculated

Our web collection on statistics for biologists contains articles on many of the points above.

Software and code

Policy information about availability of computer code

Data collection	No software was used for data collection
Data analysis	For miRNA raw sequencing reads demultiplexing : bcl2fastq 1.8.4<br>For miRNA quality control and trimming: FastQC v 0.11.5, Trimmomatic v0.36 and FastX Toolkit v0.0.14<br>For miRNA annotation and quantification: OASIS 2.0 and miRbase21<br>For total RNAseq raw sequencing reads demultiplexing : bcl2fastq 1.8.4<br>For total RNAseq quality control and trimming: FastQC v 0.11.5 and Trimmomatic v0.36,<br>For total RNAseq mapping: STAR v2.5.0<br>For total RNAseq expression quantification: cufflinks v2.2.1<br>For genotype QC, mapping and processing: Bowtie 2 v2.2.8 GATK 3.5-0 and SnpEFF-4.3.2<br>For eQTL mapping: PLINK v1.9<br>For Statistical analysis : JMP genomics v8 (SAS Institute)<br>For miRNA qPCR analysis: GeneGlobe https://geneglobe.qiagen.com/us/analyze/<br>For custom scripts: https://github.com/Yidaghdour/malaria-miRNA<br>For gene enrichment analysis: Ingenuity Pathway Analysis

For manuscripts utilizing custom algorithms or software that are central to the research but not yet described in published literature, software must be made available to editors and reviewers. We strongly encourage code deposition in a community repository (e.g. GitHub). See the Nature Research guidelines for submitting code & software for further information.

Data

Policy information about availability of data

All manuscripts must include a data availability statement. This statement should provide the following information, where applicable:

- Accession codes, unique identifiers, or web links for publicly available datasets
- A list of figures that have associated raw data
- A description of any restrictions on data availability

All the miRNAs and mRNA expression data reported in this paper were deposited in the Gene Expression Omnibus (GEO) database under accession number GSE144486 (https://www.ncbi.nlm.nih.gov/geo/query/acc.cgi?acc=GSE144486).
The genotyping dataset generated and analyzed in study is available from the corresponding author on reasonable request. Access to the data will be granted to researchers for appropriate use consistent with the consent provided by the study participants.

Field-specific reporting

Please select the one below that is the best fit for your research. If you are not sure, read the appropriate sections before making your selection.

[x] Life sciences [ ] Behavioural & social sciences [ ] Ecological, evolutionary & environmental sciences

For a reference copy of the document with all sections, see nature.com/documents/nr-reporting-summary-flat.pdf

Life sciences study design

All studies must disclose on these points even when the disclosure is negative.

Sample size	For miRNA profiling, sample size of the Discovery set is 68 samples (19 individuals in the Before Infection group, 16 in the Asymptomatic Parasitemia group, 17 in the Symptomatic Parasitemia group and, 16 in the After Treatment group). Sample size of the replication set is 53 samples from 53 individuals. In total 121 samples have been profiled in this study. For total RNA profiling , 51 samples from 51 individuals were included. Sample size was determined using power analysis as implemented in JMP genomics with the call to detect miRNA differential expression at FDR 5%. For eQTL analysis using the replication set, sample size was determined based on an average cis-eQTL effect size of 30% and 5% FDR.
Data exclusions	Four miRNA samples were excluded after bioinformatic quality control of the expression data. These four samples are not included in the sample sizes mentioned above.
Replication	This project included a replication/validation study. In total 36 miRNAs (38%) were confirmed as being differentially expressed for the infection effect (in the Discovery set) and associated with parasitemia (in the Replication set).
Randomization	The study is based on a prospective design where non-infected study participants subjects are recruited and followed over time. Therefore samples/individuals were not allocated into experimental groups. Covariates (age, sex and white blood cell count) were accounted for in ANCOVA and multiple regression models. Technical randomization of samples was done throughout the various experimental stages of the study starting with sample processing in the field to RNA extraction, library preparation, and sequencing.
Blinding	This is not an interventional study.

Reporting for specific materials, systems and methods

We require information from authors about some types of materials, experimental systems and methods used in many studies. Here, indicate whether each material, system or method listed is relevant to your study. If you are not sure if a list item applies to your research, read the appropriate section before selecting a response.

Materials & experimental systems

n/a	Involved in the study
x	Antibodies
	x Eukaryotic cell lines
x	Palaeontology and archaeology
x	Animals and other organisms
	x Human research participants
x	Clinical data
x	Dual use research of concern

Methods

n/a	Involved in the study
x	ChIP-seq
x	Flow cytometry
x	MRI-based neuroimaging

Eukaryotic cell lines

Policy information about cell lines

Cell line source(s)	HEK293FT from ThermoFisher (catalog number: R70007) and Hela cells from ATCC (catalog number ATCC CCL-2)
Authentication	None of the cell lines were authenticated.
Mycoplasma contamination	The cell lines were not tested for mycoplasma contamination.
Commonly misidentified lines (See ICLAC register)	No commonly misidentified cell lines were used in the study.

Human research participants

Policy information about studies involving human research participants

Population characteristics	Ethnicity: Gouin ethnic group in Burkina Faso, age: 2-10 years, sex: males and females. Clinical and phenotypic characteristics are provided in Supplementary Table S1.
Recruitment	Participants were recruited in the Banfora health district following approved IRB protocols. The community of the health district was informed about the study by the Centre National de Recherche et Formation sur le Paludisme (CNRFP). Subsequently, study participants were recruited in an unbiased way but following exclusion and inclusion criteria listed in the method section of the paper.
Ethics oversight	The study was approved by the Ethical Committee of the Ministry of Health of Burkina Faso (Ministry of Health, Burkina Faso; protocol number 2015-02-018) and the Institutional Review Board of New York University Abu Dhabi (UAE, protocol number 011-2015).

Note that full information on the approval of the study protocol must also be provided in the manuscript.
