# Supplementary material for: Integrative genomic analysis reveals mechanisms of immune evasion in P. falciparum malaria
Source: Nat Commun. 2020 Oct 9;11:5093. doi: 10.1038/s41467-020-18915-6 (PMC7547729; doi:10.1038/s41467-020-18915-6)
Supplement: Supplementary file 4 — Description of Additional Supplementary Files [file 41467_2020_18915_MOESM4_ESM.pdf]

**Description of Additional Supplementary Files**

File Name: Supplementary Data 1
Description: Characteristics of the study participants in the discovery (sheet a) and replication (sheet b) sets. Description of variables is provided in the sheet named “LEGEND”.

File Name: Supplementary Data 2
Description: Raw and trimmed read counts for all human small RNAs detected in the discovery set (n = 68, sheet a) and replication (n = 53, sheet b). Description of columns headers is provided in the sheet “LEGEND”.

File Name: Supplementary Data 3
Description: Results of miRNA differential expression analysis using analysis of covariance (Benjamini-Hochberg FDR < 5% for statistical significance) for the discovery set (n = 68) in sheet a. Sheet b corresponds to the results of the miRNA and log2 parasitemia association analysis in the replication set (multiple regression, Benjamini-Hochberg FDR < 5% for statistical significance). Description of columns headers is provided in the sheet “LEGEND”.

File Name: Supplementary Data 4
Description: miRNA quantitative PCR validation data (n = 39). Sheet a: Magnitude of differential expression (log2 FC) of 16 miRNAs obtained from qPCR (two-tailed Student’s t-test, P < 0.01) and the NGS analysis. Description of columns headers is provided in the sheet “LEGEND”.

File Name: Supplementary Data 5
Description: Results of miRNA-mRNA correlation analysis. Sheet a: Expression data of 133 miRNAs were tested for association against expression data of 12,375 mRNA obtained from the same set of individuals in the replication set (n = 51). Statistical significance for Pearson correlation was determined based on a nominal P threshold of 5%). Description of columns headers is provided in the sheet “LEGEND”.

File Name: Supplementary Data 6
Description: Genes enrichment analysis. Sheet a: Results of canonical pathway analysis obtained from the analysis of 456 unique genes targeted by 88 miRNAs. Significance was assessed using right-tailed Fisher's exact test (P < 0.01). Sheet b: Categories enriched obtained from the same 456 genes correlated with miRNA (right-tailed Fisher's Exact Test, BenjaminiHochberg P < 0.05). Sheet c: Statistical significance of the Cell Death and Survival subcategories enriched (right-tailed Fisher's Exact Test). Description of columns headers is provided in the sheet “LEGEND”.

File Name: Supplementary Data 7
Description: Upstream regulator analysis. Sheet a: miRNAs identified as upstream regulators and their target genes expressed in our samples. (Fisher's Exact Test, P < 0.05 for statistical significance). Description of columns headers is provided in the sheet “LEGEND”.

File Name: Supplementary Data 8
Description: cis miR-eQTL associations. Sheet a: Results of association mapping for 276 miRNAs from 46 individuals (Replication set) using 129,310 cis-SNPs and a multiple regression model (PLINK v1.9). BenjaminiHochberg FDR (5%) and 100,000 permutations test were used to assess statistical significance. Description of columns headers is provided in the sheet “LEGEND”.
